# Ankylosed Primary Molar in a Japanese Child with Hypophosphatasia

**DOI:** 10.3390/dj9010003

**Published:** 2020-12-29

**Authors:** Masakazu Hamada, Rena Okawa, Saaya Matayoshi, Yuko Ogaya, Ryota Nomura, Narikazu Uzawa, Kazuhiko Nakano

**Affiliations:** 1 Department of Oral and Maxillofacial Surgery II, Osaka University Graduate School of Dentistry, Osaka 565-0871, Japan; hmdmskz@dent.osaka-u.ac.jp (M.H.); uzawa@dent.osaka-u.ac.jp (N.U.); 2 Department of Pediatric Dentistry, Osaka University Graduate School of Dentistry, Osaka 565-0871, Japan; matayoshi@dent.osaka-u.ac.jp (S.M.); ogaya@dent.osaka-u.ac.jp (Y.O.); rnomura@dent.osaka-u.ac.jp (R.N.); nakano@dent.osaka-u.ac.jp (K.N.)

**Keywords:** hypophosphatasia, ankylosis, disturbed cementum formation, early exfoliation, extraction

## Abstract

Hypophosphatasia (HPP) is a rare genetic disorder; affected patients may experience early exfoliation of primary teeth, especially anterior teeth. However, there have been few reports regarding longitudinal follow-up for primary teeth, especially posterior teeth, until their replacement with permanent teeth. Here, we describe a patient with HPP who underwent follow-up from 1 to 9 years of age. A 14-month-old boy was referred to our hospital with the chief complaint of early loss of primary anterior teeth. He was diagnosed with odonto-type HPP by his pediatrician, due to low serum alkaline phosphatase concentration and early exfoliation of primary teeth with bone hypomineralization. The patient experienced exfoliation of three additional primary anterior teeth by 4 years and 1 month of age. Partial dentures were applied for space maintenance; there were no problems regarding subsequent replacement with permanent teeth in the anterior region. However, the primary mandibular right first molar appeared to be submerged when the patient was 8 years and 3 months of age; the severity of submergence was greater when the patient was 9 years of age. The affected primary molar was considered to be ankylosed; it was extracted when the patient was 9 years and 4 months of age. Histopathological analysis of the tooth revealed disturbed cementum formation, which is a typical characteristic of teeth in patients with HPP. On the basis of these findings, we hypothesize that the disturbed cementum formation could lead to susceptibility to early exfoliation of anterior teeth, as well as occurrence of ankylosis involving posterior teeth.

## 1. Introduction

Hypophosphatasia (HPP, OMIM entry #241500) is a rare genetic disorder, characterized by bone hypomineralization and rickets-like changes in radiological examinations, as well as reduced serum alkaline phosphatase concentrations in blood tests [1,2,3,4,5]. HPP has a wide range of onset age and severity, and is usually classified into the following six types: perinatal severe (fetal-neonatal, characterized by respiratory insufficiency and hypercalcemia); perinatal benign (fetal-neonatal, characterized by prenatal skeletal manifestations that slowly resolve into a milder form); infantile (age < 6 months, characterized by rickets and an absence of elevated serum alkaline phosphatase activity); childhood (age ≥6 months to <18 years; characterized by manifestations that range from low bone mineral density for age (with unexplained fractures) to rickets (with premature loss of primary teeth with intact roots); adult (age ≥ 18 years, characterized by stress fractures and pseudofractures of lower extremities in middle age); and odonto (regardless of age, characterized by premature exfoliation of primary teeth without skeletal manifestations) [1,2,3,4,5].

Typical dental manifestations of HPP are known to include early exfoliation of primary incisors before 4 years of age [6,7,8]. This manifestation sometimes leads to diagnosis of mild HPP (e.g., odonto and childhood types) [9,10]. To the best of our knowledge, there have been few reports regarding long-term follow-up in patients with dental manifestations of HPP after early exfoliation of primary incisors [11,12,13]. Here, we describe a patient with HPP who completed longitudinal follow-up for both posterior and anterior primary teeth. We also describe a new finding in primary teeth and provide our hypothesis regarding the pathogenesis of ankylosis in the teeth of patients with HPP.

## 2. Case Report

A 14-month-old boy was referred to the Department of Pediatric Dentistry at Osaka University Dental Hospital with the chief complaint of early loss of primary teeth. Intraoral examinations revealed that the primary mandibular bilateral incisors and the primary mandibular left lateral incisor were missing. We previously described a patient with HPP who was diagnosed on the basis of early exfoliation of primary teeth [9]. The present patient exhibited a low level of serum alkaline phosphatase (192 IU/I); radiological examinations of the lower extremities and hands did not show metaphyseal irregularities indicative of rickets. Tissue-nonspecific alkaline phosphatase gene sequencing demonstrated a heterozygous c.550C > T (p.Arg184Trp) mutation. The patient’s condition was classified as odonto-type HPP, based on the results of these examinations.

**Figure 1:**
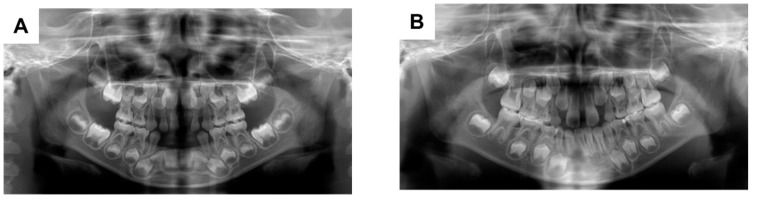

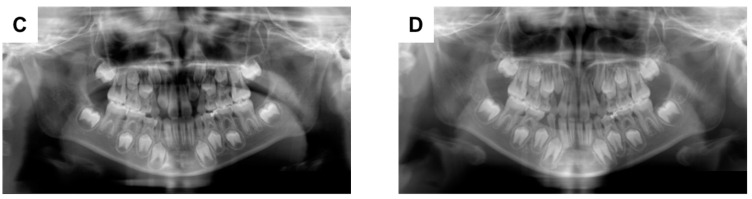
Orthopantomographs taken during follow-up. When the patient was aged (**A**) 5 years and 10 months; (**B**) 8 years and 3 months; (**C**) 9 years; and (**D**) 10 years and 1 month.

**Figure 2:**
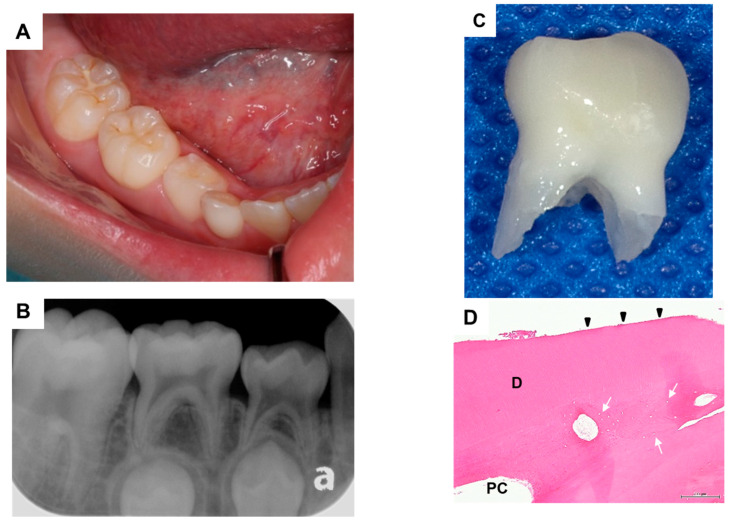
(**A**) Intraoral photograph taken before tooth extraction; (**B**) Dental radiograph taken before tooth extraction; (**C**) Extracted tooth; (**D**) Histopathological appearance of exfoliated molar. Disturbed cementum formation (arrowhead) and acellular cementum (arrow) are visible. D, dentin and PC, pulp chamber.

The patient’s primary mandibular right lateral incisor spontaneously exfoliated when he was 2 years and 11 months of age; the primary maxillary left incisor also exfoliated when he was 4 years and 1 month of age. Partial dentures were applied for space maintenance; there were no problems regarding subsequent replacement with the corresponding permanent teeth (Figure 1A). However, the primary mandibular right first molar was appeared to be submerged when the patient was 8 years and 3 months of age; the severity of submergence was greater when the patient was 9 years of age (Figure 1B,C). The primary mandibular right first molar was about 1.5 mm submerged from the primary mandibular right second molar, and the periodontal ligament cavity was partially unclear (Figure 2A,B). The primary mandibular right first molar was considered to be ankylosed, although the corresponding tooth in the opposite quadrant did not show similar findings. Thus, extraction of the affected tooth was performed (Figure 2C). Histopathological analysis of the tooth showed disturbed cementum formation and acellular cementum, with wide and wavy ivory tubules and aplasia (Figure 2D). The patient’s postoperative course was good, and no particular complications were observed. The mandibular right mandibular first premolar showed a tendency to erupt (Figure 1D).

## 3. Discussion

Early exfoliation of primary teeth is a well-known dental manifestation in patients with HPP [6,7,8,9,10]. Early exfoliation is presumably limited to the anterior region because of characteristics such as the time of eruption, tooth morphology, and number of roots involved. The primary incisors erupt first in primary dentition; these influence occlusal force. The primary incisors are also short; each exhibits a single, small root. However, there have been no reports regarding primary molars in patients with HPP who experience early exfoliation of primary incisors.

Here, we described a patient with HPP who completed a longitudinal follow-up period of approximately 10 years. During follow-up, the primary mandibular right first molar was submerged when the patient was 8 years of age; this submergence demonstrated further progression when the patient was 9 years of age. Therefore, the primary mandibular right first molar was considered to be ankylosed. We extracted a lower position of the patient’s primary teeth. Histopathological analysis of extracted tooth revealed disturbed cementum formation. Then, we confirmed the diagnosis as ankylosis. Thus, we presumed that ankylosis had been caused by disturbed cementum formation and altered occlusal force. The poor periodontal condition (due to disturbed cementum formation related to HPP) led to the absence of a root connection with alveolar bone by means of periodontal ligament. The excessive occlusal force gradually damaged this periodontal tissue and subsequently affected root adhesion to alveolar bone.

**Figure 3:**
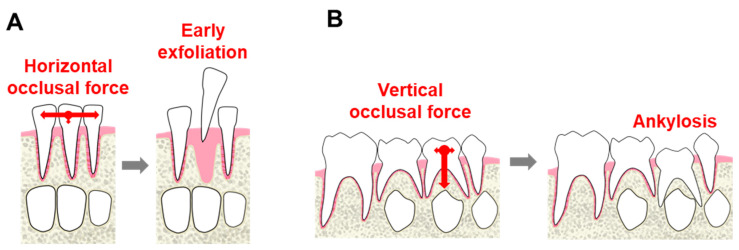
Possible mechanism of ankylosis. (**A**) In anterior teeth, lateral force may be greater than vertical force and lateral force can promote exfoliation of teeth; (**B**) Occlusal force acts vertically in molars and may resist exfoliation involved in ankylosis.

We suspect that the following mechanism contributed to the pathogenesis of ankylosis in this patient. In anterior teeth, lateral occlusal force is stronger than vertical force (Figure 3A); this lateral force can promote tooth exfoliation. Conversely, occlusal force acts vertically in the posterior region, such that affected teeth may resist exfoliation (Figure 3B). Therefore, ankylosis may occur in molars, but is less likely to occur in anterior teeth. Although this is the first report of ankylosis in a patient with HPP, the contributing factors are not fully clear and data for additional patients are needed; however, in patients with other severe types of HPP, exfoliation and ankylosis may not occur.

Notably, ankylosis was detected only in the primary mandibular right first molar. The opposite first molar was filled with composite resin due to caries; and the site of restoration was adjusted to reduce occlusion pressure. Alternatively, the partial denture clasp may have exerted excessive vertical force on the primary mandibular right first molar. The reason for local occurrence of ankylosis is unclear; thus, we plan to observe dentition exchange carefully in this patient.

Partial dentures are used for space maintenance in patients with HPP who experience early exfoliation [6,9]. In our patient, a mandibular denture was applied at the age of 4 years and a maxillary denture was applied at the age of 5 years; subsequent tooth replacement did not show any problems. We also considered the use of a band loop appliance for space maintenance after extraction of this ankylosed primary first molar; however, we did not apply this appliance because of the high likelihood of early exfoliation involving the mandibular right second molar. The patient’s orthodontist noted that the primary mandibular first and second molars would not move in the mesial direction because the mandibular first molar had fully erupted; distal movement of primary canines was desirable to resolve the patient’s tendency for anterior crossbite.

Tooth movement is more likely to occur in patients with HPP, because they exhibit weaker external force than the general population. Therefore, care is needed when performing an oral surgical procedure (e.g., tooth extraction) in these patients, to avoid application of excessive force to adjacent teeth.

## 4. Conclusions

Early exfoliation and ankylosis are important considerations in patients with HPP. Disturbed cementum formation in patients with HPP leads to early exfoliation in the anterior region and ankylosis in the posterior region. Ankylosis in molars might be caused by vertical occlusal force acting on periodontal tissue; early exfoliation in incisors might be caused by horizontal occlusal force. Dentists should focus on dentition and occlusion in primary dentition of patients with HPP. However, since HPP is a rare disease, there are many unclear points, and therefore it is necessary to accumulate cases. Total oral management should be performed with consideration of the possibility of ankylosis, as well as attention to premature tooth loss, during dental treatment of patients with HPP.

## Data Availability

Not applicable.
